# OD24 Scalability Analysis Of Multimodal Prehabilitation For Frail Elderly Patients Before Elective Surgery In Germany

**DOI:** 10.1017/S0266462324001569

**Published:** 2025-01-07

**Authors:** Susanne Felgner, Helene Eckhardt, Zoe Weber, Wilm Quentin, Tanja Rombey

## Abstract

**Introduction:**

Multimodal prehabilitation, including interventions like physiotherapy, combined with frailty screening and a shared decision-making conference for frail elderly patients before elective surgery is an innovative approach currently under investigation (PRAEP-GO RCT, NCT04418271). The PRAEP-GO intervention aims to enhance postoperative outcomes and prevent care dependency. Our aim is to systematically assess the scale-up potential of PRAEP-GO within the German healthcare system.

**Methods:**

We are conducting a scalability analysis using the Intervention Scalability Assessment Tool (ISAT). The ISAT questionnaire comprises two parts: (A) “Setting the scene,” describing the current health service situation (e.g., intervention characteristics, political context), and (B) “Intervention implementation planning,” outlining future requirements (e.g., workforce, infrastructure), with open-ended questions and a scalability readiness assessment using a scale. Our analysis involves three stages: (i) health economists from the PRAEP-GO research team individually answering ISAT questions, (ii) trialists from the PRAEP-GO research team interviewed in a group, and (iii) external experts representing relevant stakeholders for future implementation interviewed in an advisory board meeting.

**Results:**

Data collection for stage (i) and (ii) has been completed, while data collection for stage (iii) is expected to be completed in February 2024. The preliminary findings for part (A) highlight the need for a sustainable approach to manage an aging and increasingly frail patient population requiring surgery. There is no clinical guideline available for the management of this population group. Regarding part (B), the current infrastructure (e.g., therapy facilities) and personnel structures might need to be adapted and should be expanded for large-scale application. Employing professionals to coordinate the patient pathway was recommended, along with adjustments to reimbursement structures.

**Conclusions:**

In PRAEP-GO, we are pursuing a multidisciplinary process with the aim of supporting health decisions that promote an equitable, efficient, and high-quality healthcare system meeting the challenges of an aging population due to demographic change. The PRAEP-GO trial is currently exploring this approach on a small scale. Existing infrastructure and personnel structures would need to be adapted and expanded for scale-up.

